# The effect of macronutrient and micronutrient supplements on COVID-19: an umbrella review

**DOI:** 10.1186/s41043-024-00504-8

**Published:** 2024-01-29

**Authors:** SeyedAhmad SeyedAlinaghi, Ramin Shahidi, Hengameh Mojdeganlou, Fatemeh Khajeh Akhtaran, Seyed Farzad Maroufi, Seyede Parmis Maroufi, Pegah Mirzapour, Amirali Karimi, Sepideh Khodaei, Mehrzad Mohsseni Pour, Esmaeil Mehraeen, Omid Dadras

**Affiliations:** 1 Iranian Research Center for HIV/AIDS, Iranian Institute for Reduction of High Risk Behaviors, Tehran University of Medical Sciences, Tehran, Iran; 2 School of Medicine, Bushehr University of Medical Sciences, Bushehr, Iran; 3 Department of Pathology, School of Medicine, The Johns Hopkins University, Baltimore, MD USA; 4 Social and Economic Statistics, Faculty of Mathematical Sciences, Shahid Beheshti University, Tehran, Iran; 5 School of Medicine, Tehran University of Medical Sciences, Tehran, Iran; 6 Faculty of Medicine, Semnan University of Medical Sciences, Semnan, Iran; 7 Department of Health Information Technology, Khalkhal University of Medical Sciences, Khalkhal, 5681761351 Iran; 8 Bergen Addiction Research, Department of Addiction Medicine, Haukeland University Hospital, Bergen, Norway

**Keywords:** COVID-19, Macronutrients, Micronutrients, Nutrition, Nutrients, SARS-CoV-2

## Abstract

**Background and aims:**

A healthy diet play an important role in the prevention and even treatment of various diseases. Proper nutrition plays an important role in boosting of immune system. These include the consumption of macronutrients such as proteins, lipids, carbohydrates, and also micronutrients including vitamins. Here, we aimed to systematically review the effects of macronutrients and micronutrients on the prevention and treatment of COVID-19.

**Methods:**

We searched the databases of PubMed, Scopus, Embase, and Web of Science on December 23, 2023. The records were downloaded into an EndNote file, the duplicates were removed, and the studies underwent a two-phase screening process based on their title/abstracts and full texts. The included articles were screened and underwent inclusion and exclusion criteria. We included the English systematic reviews and meta-analyses that concurred with the aim of our study. The selected articles were assessed by Cochrane's Risk of Bias in Systematic Reviews for the quality check. The data of the eligible studies were extracted in a pre-designed word table and were used for the qualitative synthesis.

**Results:**

A total of 28 reviews were included in this study. Most studies have shown that micronutrients are effective in morbidity and mortality controlling in viral respiratory infections such as COVID-19 but some studies have shown that micronutrients are sometimes not effective in controlling severity. On the other hand, calcifediol was by far the most successful agent in reducing intensive care needs and mortality between studies.

**Conclusion:**

Individuals without malnutrition had a reduced risk of SARS-CoV-2 infection and severe disease. The administration of Vitamin D is effective in reducing the morbidity and mortality of COVID-19 patients. Patients with vitamin D deficiency were more prone to experience severe infection, and they were at higher risk of morbidities and mortality. Other micronutrients such as Vitamin A, Vitamin B, and Zinc also showed some benefits in patients with COVID-19. Vitamin C showed no efficacy in COVID-19 management even in intravenous form or in high doses.

**Supplementary Information:**

The online version contains supplementary material available at 10.1186/s41043-024-00504-8.

## Introduction

In recent years, COVID-19 has been one of the most challenging global issues and all efforts are being made to control this disease [1, 2]. One effective way to reduce the risk of viral infections is to have a healthy diet, exercise, and lifestyle [3, 4]. Proper nutrition includes the consumption of macronutrients such as proteins, lipids, and carbohydrates, as well as micronutrients such as vitamins found in food and water (minerals) [5]. Nutrition plays an important role in the immune system. Of course, for better results, certain nutrients should be added as sources of antioxidants, such as fresh vegetables, fruits, nuts and soy, and omega-3 fatty acids [5]. In fact, nutrition can play an important role in any stage of the disease. In the pre-disease period, it is important for prevention, and during infection, the immune system requires almost 30% of the calories consumed by basal metabolism (BM). Therefore, the immune function of people with inadequate macronutrient intake is likely to be compromised and their susceptibility to infection increased. The results of sufficient energy intake on immune system function have been observed previously. So that in the infection stage, it is very useful for boosting the immune system. In areas where there is nutrient deficiency, it lowers the immune system and increases mortality [6, 7].

Energy-deficient diet also increases the risk of consuming inadequate amounts of micronutrients. Micronutrients play a key role in mediating inflammatory responses and modulating chronic inflammatory diseases. Suboptimal micronutrient status increases the risk of contracting and duration of infectious diseases. Micronutrient deficiencies can reduce cytokine responses and immune cell mediated responses to pathogens. Disruption in the regulation of immune homeostasis may occur in conditions of deficiency of zinc and copper due to the negative effect on the number and function of immune cells. Micronutrient status can influence the genetic makeup of a viral pathogen, thereby contributing to the development of new ones [7].

The use of minerals and vitamins plays an important role in improving the immune system and thus improving COVID-19 disease [8]. Vitamins have antioxidant properties and immunomodulatory effects, and minerals are involved in physiological processes such as bone growth, heart rate regulation, blood formation, and hormone synthesis [9]. They are also involved in epigenetic symptoms such as histone modification, DNA methylation, and post-translational changes [10]. Minerals such as zinc, iron, magnesium, copper, and selenium are very effective in strengthening the immune system [8]. Selenium in the form of amino acid, selenocysteine, form part of the catalytic site of peroxidases, which play a role in antioxidant defense, and redox homeostasis during viral disease. Since viral infections produce oxidative stress, selenoproteins are critical for host defense. In the case of selenium deficiency (SD), selenoprotein expression decreases and facilitates increased oxidative stress, which can lead to viral mutation, and increased pathogenicity. SD is also associated with increased pathogenicity of some viruses such as influenza, and supplementation may reverse or reduce the risk of other pathologies associated with oxidative stress, such as infections [7].

Vitamins C and D, iron and zinc can support immune system function. So that vitamin D has immunomodulatory effects on cells of the innate and adaptive immune system through endocrine mechanisms. Most immune cells have a vitamin D receptor and its activating enzyme, 1-α-hydroxylase, and vitamin D have been shown to protect the skin, respiratory, and digestive tracts. Vitamin D supports the physical barrier by upregulating mRNA production of the antimicrobial peptide, cathelcidin, to enhance clearance of bacteria at epithelial cells. When vitamin D status is sufficient, upregulation of vitamin D receptor and 1-α-hydroxylase expression activates vitamin D in human macrophages during pathogen invasion. This in turn regulates the expression of cathelcidin, which is involved in the innate immune response to bacterial infection. Deficiency is associated with higher susceptibility to infection by microbial infections [7].

Protein intake is in relation with cellular immunity. In cases of protein-energy malnutrition, often observed in low-income countries, immune function is impaired, with reduced epithelial and physiological function, and impaired function of macrophages, neutrophils, and natural killer cell. In the other hand, atrophy of lymphatic organs and T-lymphocyte deficiency are seen with protein deficiency, which increases susceptibility to viral infections [7].

Proteins and amino acids also support the body's homeostasis. Adequate protein intake is important to provide adequate energy and reduce infectious complications for critically ill patients [11]. When the immune system is activated by pathogens, it increases the demand for glucose, amino acids, and fatty acids, so with good and timely nutrition of vitamins, minerals, and proteins, the body can be protected against pathogens [8]. Finally, it should be known that the use of micronutrients and macronutrients is a relatively cheap and easy treatment to manage the process of controlling COVID-19 [12].

One effective way to reduce the risk of COVID-19 is to have a healthy diet. Many studies have been conducted, but there are so conflicting findings that can be due to selection or information biases. Thus, focusing on studying systematic reviews can earn stronger scientific evidence and subsequently resolve the contradictory results. Therefore, the authors aim to perform an umbrella review on the role of micronutrients and macronutrients in COVID-19.

## Methods

This umbrella review of systematic reviews was conducted on December 23, 2023, to study the effects of macronutrients and micronutrients on COVID-19. The main purpose of this study is to investigate the effect of macronutrient and micronutrient supplements on COVID-19.

### Search sources

We searched the databases of PubMed, Scopus, Embase, and Web of Science on December 23, 2023.

### Search characteristics

First, we selected a detailed list of keywords using the medical subject headings (MeSH) website, Embase’s Emtree, and the keywords of the previously published studies. We then developed a search strategy using these keywords and searched the databases. A detailed search strategy for each database is recorded in the Additional file 1.

### Screening and inclusion/exclusion criteria

We included systematic reviews and meta-analyses on the role of micronutrients and macronutrients in the prevention or severity of COVID-19. Only articles that were aligned with the purpose of the present study and were written according to the standards of a systematic review were included.

The exclusion criteria were the following:

1. Studies other than systematic reviews and meta-analyses, including original studies and non-systematic reviews
2. Abstracts, conference abstracts, and studies without available full texts
3. Studies not related to nutrients, or not related to the COVID-19
4. Non-English studies

Regarding the screening process, we first downloaded the records that appeared in the database search to the EndNote application and removed the duplicates. Then, two researchers screened the studies based on the cohesion of their title and abstract to the inclusion criteria. Then in the second phase, the studies were screened by their full texts, and the eligible studies entered the qualitative synthesis. In case of any disagreements, the authors sought the opinion of a third researcher.

### Data extraction

Three independent researchers extracted the relevant data from the included review studies and organized them in a table. The data included the name of authors, study objectives, the regions of the study, the number of the included studies, the types of the included studies, the checklists that had been used for quality assessment, the databases that had been searched, and the main findings, including the role of macronutrients and micronutrients on the COVID-19 (Table 1).

**Table 1:** Identified the macronutrient and micronutrient supplements

ID	The first author (reference)	Year	Objectives of study	Number of included studies	Type of included studies	Quality assessment tools	Type of databases	Main result/finding
1	Mazidimoradi, Afrooz. [13]	2022	Effect of PUFA on COVID-19 patients	18	Prospective, Retrospective cohort, Retrospective, Cross-sectional, Clinical trial, Cross sectional	Adapted Newcastle–Ottawa Quality Assessment Scales	PubMed, Scopus, and Web of Science	Omega 3 PUFA intake reduces the risk and severity of COVID-19 Omega 3 and Omega 6 PUFAs level are lower in patients with COVID-19 Upper levels of PUFAs decrease the need for mechanical ventilation and hospitalization in COVID-19
2	da Rocha, Aline Pereira. [14]	2021	To assess whether vitamin D supplementation is safe and effective for the treatment of COVID-19	3	Clinical trial	GRADEpro platform	Medline, PubMed, Embase, Elsevier, Cochrane Central	Vitamin D has no effect on mortality rate, inflammatory markers, and duration of invasive mechanical ventilation in COVID-19 patients Vitamin D plus standard usage can lower the risk of ICU admission
3	Sharma, L. [15]	2020	Nutritional supplementation for immunity against COVID-19	12	Case study, Review	NA	Scopus, Pub Med, Web of science	Vitamin A enhances T cells and B cells activation and has anti-inflammatory effects vitamin C prevents and treats respiratory and systemic infections and improves the activities of the immune system. Vitamin E has various advantages for the immunity system. Vitamin B9 increases body resistance against infectious diseases. Zinc regulates our immunity. Copper is essential for the growth, metabolism of iron, functioning of neuroendocrine, elasticity of lungs, cardiovascular integrity, and neovascularization Iron is needed for the normal functioning of immune and non-immune cell reactions. Selenium improves immunity against viral infections
4	Ao, Guangyu. [16]	2022	IV vitamin C usage for treating COVID-19	7	Clinical trial, Observational studies	Jadad scale, NOS	PubMed, Embase, Cochrane Library, MEDLINE, Web of Science	Vitamin C does not affect the severity and mortality of COVID-19
5	Bassatne, Aya. [17]	2021	COVID-19 and Vitamin D	34	Observational studies, Clinical trial	New Castle-Ottawa quality scale Cochrane GRADE	Medline, Embase, Cochrane	Vitamin D lower than 20 increases mortality, ICU admission, and invasive and non-invasive mechanical ventilation Vitamin D level does not affect inflammatory markers in COVID-19
6	Nikniaz, Leila. [18]	2021	Vitamin D and COVID-19	4	Clinical trial, Quasi-experimental	JBI Critical Appraisal	PubMed, Scopus, Web of Science, Embase, Cochrane	Vitamin D decreases mortality rate, severity, and inflammatory markers
7	Kwak, Sang Gyu. [19]	2021	Therapeutic effect of high-dose intravenous vitamin C (HDIVC) in patients with COVID-19	8	Clinical trial, Retrospective studies	Cochrane Collaboration tool Newcastle–Ottawa scale	PubMed, Cochrane, Embase, and Web of Science	High-dose IV vitamin C does not significantly reduce mortality rate and length of hospitalization
8	Grove, Amy. [20]	2021	Vitamin D and COVID infection	4	Cross sectional, Retrospective cohort, Case–control	Downs and Black Quality Assessment Checklist	MEDLINE, Embase, Cochrane, MedRxiv, and BioRxiv	No association between ethnicity and vitamin D deficiency with COVID-19
9	Beran, Azizullah. [21]	2022	Micronutrient supplements and COVID infection	26	Clinical trial, Retrospective cohort, Observational,	Jadad composite scale, Newcastle Ottawa Quality Assessment Scale	PUBMED/MEDLINE, Embase, and Cochrane	Vitamin C supplements: no significant effect on mortality, intubation rate, and length of hospital stay (LOS) in COVID-19/ vitamin D supplementation: no significant effect on mortality, a significant reduction in intubation rate and LOS, Zinc supplementation: no significant effect on mortality
10	Khoiroh, Mawadatul. [22]	2021	Vitamin D in reducing the clinical impact of COVID-19	6	Clinical trial, Quasi-experimental, Cohort	Cochrane Risk of Bias Tool	Scopus, ScienceDirect, and PUBMED	Significant relationship between the administration of vitamin D and length of hospital stay, ICU stay, cure rate, severity, mortality, and signs of inflammation
11	Varikasuvu, Seshadri Reddy.[23]	2022	Evaluate the use of vitamin D intervention on COVID-19 outcomes	6	Clinical trial	Cochrane Risk of Bias Tool	PubMed, Cochrane library, and ClinicalTrials.gov	Overall outcomes: beneficial use of vitamin D intervention in COVID-19 (relative risk, RR = 0.60)/ no statistical significance was observed for individual outcomes of ICU care and mortality/ RT-CR positivity was significantly decreased in the intervention group
12	Beran, Azizullah Beran. [24]	2021	Effect of vitamin D, vitamin C, and zinc on mortality in COVID-19	16	NA	NA	PubMed, Embase, and Cochrane Library	Both vitamin C and D did not significantly reduce mortality/ zinc reduced mortality significantly
13	Lim [25]	2021	Associations between micronutrient supplementation or deficiency, with novel coronavirus incidence and disease severity	52	Cohort, Case control, Cross-sectional, Clinical trial	National Heart, Lung and Blood Institute (NHLBI) quality assessment tool	Pubmed, EMBASE, Cochrane, Scopus, and CINAHL	Without micronutrient deficiency is associated with reduced odds of COVID-19 incidence, ICU admissions, or severe/critical disease onset when combined as a severity outcome/ insignificant effect on mortality, ICU admission, progression to respiratory-related complications, severe/critical disease onset or requiring respiratory support and hospitalization rate
14	Dissanayake, Harsha Anuruddhika. [26]	2022	Association between vitamin D deficiency/insufficiency and susceptibility to COVID-19, its severity, mortality, and role of vitamin D in its treatment	76	Observational, Clinical trial	Newcastle and Ottawa scales, AUB KQ1 Cochrane tool	CINAHL, Cochrane Library, EMBASE, PubMed, Scopus, and Web of Science	Vitamin D deficiency/insufficiency increased the odds of developing COVID-19 severe disease, and death/ Vitamin D concentrations were lower in severe COVID-19 and in non-survivors/ non-significant association between vitamin D deficiency/insufficiency and death
15	Khokher, Waleed. [27]	2021	Role of high-dose vitamin C (HDVC) in reducing mortality, length of intensive care unit (ICU) stay, and length of hospital stay	4	Clinical trial, Retrospective	NA	PubMed, Embase, and Cochrane	No significant effect on mortality, length of hospital stay/ significantly increased length of ICU stay
16	Petrelli, Fausto. [28]	2021	Association between vitamin D and risk, severity, and mortality for COVID-19 infection	43	Retrospective, Observational	Newcastle–Ottawa Scale checklist	PubMed, Cochrane Library, EMBASE	Vitamin D deficiency: significantly increased risk of infection, worse severity, and higher mortality
17	Ghasemian, Roya. [29]	2021	Role of vitamin D in the COVID-19	11	Retrospective, Prospective	Newcastle–Ottawa Scale checklist	PubMed, Scopus, Embase Web of Science up	Vitamin D deficiency: Higher odds of infection, a higher chance of severe COVID-19, no change in mortality
18	James, Philip T. [30]	2021	How malnutrition across all its forms may influence both susceptibility to, and progression of, COVID-19	139	NA	NA	PubMed, EMBASE, and Clinical trial registry	NA
19	da Silva Toscano, Gislani Acásia. [31]	2021	Vitamin C and D supplementation and the severity of COVID-19	NA	Case control	National Institutes of Health (2014). Quality Assessment Tool for case–control studies	PubMed, Web of Science, Scopus, Cochrane, and ScienceDirect	Supplements of vitamins D and C are effective in reducing the severity of COVID-19
20	Decyk, Agnieszka. [32]	2022	Vitamin D in SARS-COV-2 infection	NA	NA	NA	PubMed and Scopus	Vitamin D plays an important role in the mechanisms of innate immunity in the course of acute respiratory infections
21	Bania, Angelina. [33]	2022	Therapeutic Vitamin D Supplementation Following COVID-19	11	Randomized controlled trials, Prospective and retrospective observational studies, case–control studies, and case series	RoB, MINORS	PubMed and Scopus	25(OH)D3 (calcifediol) is by far the most successful agent in reducing intensive care needs and mortality
22	Hariyanto, Timotius Ivan. [34]	2022	Vitamin D supplementation and Covid‐19 outcomes	11	ClinicalTrials	NOS	PubMed, Europe PMC and ClinicalTrials.gov	Vitamin D supplementation offers beneficial effects on COVID‐19 outcomes
23	Pal, R. [35]	2021	Vitamin D supplementation and clinical outcomes in COVID‑19	13	Prospective or retrospective, cohort or case–control design, randomized controlled trials	NOS	PubMed/MEDLINE, Scopus, and Web of Science	Vitamin D supplementation is associated with improved clinical outcomes in terms of ICU admission and/or mortality, especially in those with moderate-to-severe COVID-19 requiring hospitalization
24	Gilani, Sadaf Jamal. [36]	2022	Reduce complications of COVID-19 through vitamin D	118	Systematic Reviews and Meta-Analysis	NA	Google Scholar, PubMed, NCBI, Scopus, and Web of Science	Vitamin D attenuates COVID-19 complications via modulation of pro-inflammatory cytokines, antiviral proteins, and autophagy
25	Rawat, Dimple. [37]	2021	Vitamin C and COVID-19 treatment	6	ClinicalTrials	GRADE-PRO	PubMed, Embase, Scopus, Google Scholar	There is no benefit to prescribing vitamin C in COVID-19
26	Scarpellini, Emidio.[38]	2022	Zinc and gut microbiota in health and gastrointestinal disease under the COVID-19	NA	Original articles, reviews, meta‐analyses, and case series	NA	PubMed and Medline	Zinc is effective in modulating intestinal microbiota in gastrointestinal diseases
27	Balboni, Erica.[39]	2022	Zinc and selenium supplementation in COVID-19 prevention and treatment	22	Clinical Trials	NA	Pubmed, Scopus	Selenium supplementation does not affect COVID-19
28	Huang Y [40]	2023	summarizes the macronutrient and micronutrient requirements and therapeutic effects in critically ill patients with SARS‐CoV‐2	10	Randomized control trials	NA	PubMed, CINAHL, Web of Science, and the Cochrane	Preliminary result suggests that ω‐3 fatty acids may protect against renal and respiratory impairments. The therapeutic effects of group B vitamins and vitamin Cannot be ascertained, although intravenous vitamin C appears promising in reducing mortality and inflammation

*PUFA* Polyunsaturated fatty acid, *ICU* Intensive care unit, *IV* Intra Venous, *HDIVC* High-dose intravenous vitamin C, *LOS* Length of hospital stay, *RT-PCR* Reverse transcription polymerase chain reaction, National Heart, *NHLBI* Lung and Blood Institute, *HDVC* High-dose vitamin C, *JBI* Joanna Briggs Institute, *ROB* Risk of bias, MINORS Methodological Index For Non-Randomized Studies

### Quality assessment

We used the Cochrane’s Risk of Bias in Systematic Reviews (ROBIS) for the quality assessment of our study. The tool is completed in three phases: (1) assess relevance (optional), (2) identify concerns with the review process, and (3) judge the risk of bias in the review. Signaling questions are included to help assess specific concerns about potential biases with the review. The ratings from these signaling questions help assessors to judge the overall risk of bias [41]. Table 2 summarizes the results of Cochrane’s ROBIS quality assessment tool for our study in phases 2 and 3 domains.

**Table 2:** Cochrane’s ROBIS quality assessment tool

Reference	Phase 2				Phase 3
	1. Study eligibility criteria	2. Identification and selection of studies	3. Data collection and study appraisal	4. Synthesis and findings	Risk of bias in the review
[13]	✓	✓	✓	✓	✓
[14]	✓	✓	✓	✓	✓
[15]	×	✓	✓	✓	×
[16]	✓	✓	✓	✓	✓
[17]	✓	✓	✓	✓	✓
[18]	✓	✓	✓	✓	✓
[19]	✓	✓	✓	✓	✓
[20]	✓	✓	✓	✓	✓
[21]	✓	×	✓	×	✓
[22]	✓	✓	✓	✓	✓
[23]	✓	✓	✓	✓	✓
[24]	✓	✓	✓	✓	×
[25]	✓	✓	✓	✓	✓
[26]	✓	✓	✓	✓	✓
[27]	✓	×	✓	✓	✓
[28]	×	✓	✓	✓	✓
[29]	✓	✓	✓	✓	✓
[30]	✓	✓	✓	✓	✓
[31]	✓	✓	✓	✓	✓
[32]	✓	✓	✓	✓	×
[33]	✓	×	✓	✓	✓
[34]	✓	✓	✓	✓	✓
[35]	✓	✓	✓	✓	✓
[36]	✓	✓	✓	✓	✓
[37]	✓	✓	✓	×	✓
[38]	×	✓	✓	✓	✓
[39]	✓	✓	✓	✓	✓

✓ = low risk, ×  = unclear risk

### Qualitative synthesis

We used the ROBIS method for the qualitative classification of articles. For this purpose, as shown in Table 2, the articles were categorized and selected based on criteria including “study eligibility”, “identification and selection”, “data collection and study appraisal”, and “synthesis and findings”. In the next phase, we assess the articles by the “risk of bias in the review” criteria.

### Ethics

The ethical concerns for publishing the secondary studies are considered especially primary authors' rights, copyright, and plagiarism.

## Results

**Fig. 1:**
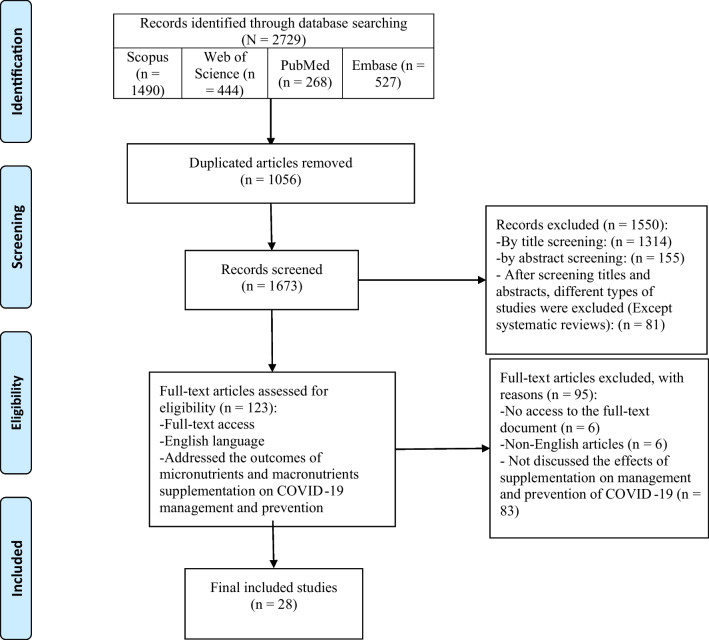
PRISMA flow diagram of this study’s selection process

The database search revealed 2729 records, of which 1056 duplicates were removed and 1673 records were screened. Then, 1550 studies were excluded in the title and abstract screening, and 123 articles entered the full-text screening. After the full-text screening, 95 studies were excluded and 28 eligible reviews were selected for qualitative synthesis in this review (Fig. 1). These systematic reviews included 3 to 139 articles and utilized different quality assessment tools for rating the quality of included studies such as the Newcastle and Ottawa scale, The Grading of Recommendations Assessment, Development and Evaluation (GRADE-PRO), and National Heart, Lung, and Blood Institute (NHLBI) assessment tool. Due to the COVID-19 pandemic and concerns about its severity and mortality [42, 43], multiple recommendations for its therapy were suggested. These medications and interventions were used on COVID-19 patients and assessments were performed.

All the studies included in this review were systematic reviews concerning the effect of macronutrient and micronutrient supplements in patients with COVID-19. All the studies were conducted between 2020 and 2023. The number of included studies, type of included studies, the quality assessment instrument for included studies, type of databases, and main results/findings were reviewed for these 28 articles.

The selected systematic reviews included a total number of 650 studies (although several studies have been repeated across the included reviews), including prospective and retrospective cohorts, cross-sectional studies, clinical trials, case series, experimental studies, reviews, systematic reviews, and meta-analyses. Newcastle and Ottawa scale (NOS) was the main instrument used to appraise the primary studies and the rating of quality [16, 34, 35]. PubMed, Scopus, Web of Science, Medline, Embase, Elsevier, Cochrane Central, Scopus, Cochrane Library, MedRxiv, BioRxiv, ScienceDirect, ClinicalTrials.gov, CINAHL, and Google Scholar are the databases searched by the studies.

Although most studies have shown that micronutrients are effective in controlling viral respiratory infections such as COVID-19, several studies have shown that micronutrients are sometimes not effective in controlling severity [16, 19, 20, 23, 24, 37, 39]. On the other hand, 25 (OH)D3 (calcifediol) was by far the most successful agent in reducing intensive care needs and mortality across the studies [33]. The results of similar studies showed that minerals also appeared to be effective in the intensity of COVID-19 [13, 15, 21, 38].

## Discussion

In this study, we included 28 review studies, which assessed the effect of macronutrient and micronutrient supplements on COVID-19. Based on the findings of the present study, vitamin D is by far the most studied micronutrient, assessed by 16 included articles [14, 18, 20–24, 26, 28, 29, 31–36]. A systematic review by Petrelli et al. evaluated 43 articles about the effect of vitamin D on COVID-19 outcomes. They discovered that vitamin D deficiency increases the risk of COVID-19 infection and its severity & mortality rate [28]. A similar study by Dissanayake including 76 studies reported that both vitamin D insufficiency and deficiency result in severe COVID-19 disease and higher mortality. They also found that patients with severe disease have lower levels of vitamin D [26]. Pal et al. showed that individuals with severe COVID-19 course benefit from vitamin D administration. It improves their disease outcome and reduces the risk of ICU admission and mortality rate [35].

Reviewing 118 studies, Gilani et al. discovered that vitamin D reduces COVID-19 complications by modulating the pro-inflammatory cytokines [36]. Hariyanto et al. also assessed the effect of vitamin D as a supplement, including 13 studies. The findings demonstrated the beneficial effects of vitamin D on COVID-19 outcomes and acknowledge the previous findings [34]. Another consistent systematic review including six studies conducted by Khoiroh et al. reported similar findings indicating the reduction of length of hospital stay (LOS), severity, and mortality, in line with Gilani’s study. Moreover, they found reduced inflammatory markers due to vitamin D administration [22, 36].

The reduction in mortality rate and severity of disease following vitamin D administration is also documented in Nikniaz’s study, in which four clinical trials and experimental studies were included [18]. Although there are multiple studies indicating that vitamin D administration can reduce the severity and mortality of the disease, they are other research showed contrary findings. For example, the study by da Rocha et al. reported that vitamin D does not affect the mortality rate; even though, it could lower the ICU admissions. It is important to mention [14]. However, these findings were also confirmed by Grove et al. review, including 4 studies. Grove et al. used the Downs and Black quality assessment checklist and found no association between vitamin D and the severity and mortality of COVID-19 [20].

Another systematic review including 26 studies evaluated the effect of vitamin C, vitamin D, and Zinc and found no association between the administration of these micronutrients and COVID-19 mortality. Only a probable association of vitamin D administration with shorter hospital stays and reduced intubation was reported [21]. Including 11 studies to evaluate the vitamin D efficacy, Ghasemian et al., found no association between vitamin D administration and COVID-19 mortality rate. Although they found that its deficiency leads to an increased risk of COVID-19 infection and severe disease course [29]. Although there are inconsistencies across studies that investigated the efficacy of micronutrients on COVID-19 infection, the majority observed that vitamin D has beneficial effects on COVID-19 morbidity and mortality.

The effects of other micronutrients are also assessed by multiple research projects including vitamin A, vitamin B, vitamin C, vitamin E, Zinc, Iron, and Selenium. Sharma et al. conducted a review study on these micronutrients including 12 reviews and case studies. The results showed that vitamin A intensifies Immune cell activation including B cells and T cells. It also has anti-inflammatory effects. The researchers also found that vitamin C has preventive effects on respiratory infections and enhances immune system activation. Sharma also reported that vitamin B9, vitamin E, Iron, and selenium enhance the immune system [15]. In contrast, Balboni evaluated 22 clinical trials and found no efficacy of selenium administration on COVID-19 outcomes [39].

One of the challenging and controversial matters in COVID-19 treatment during the pandemic was the effect of vitamin C on COVID-19 infection. Beran et al. evaluated 16 articles concerning the effect of vitamin C, vitamin D, and Zinc administration on COVID-19 outcomes. It appeared that administrating vitamin D and vitamin C in COVID-19 treatment does not affect mortality; however, a remarkable reduction in the death rate following the Zinc administration was observed [24]. A similar study by Rawat also reported no benefit for vitamin C administration in COVID-19 [37]. In contrast, da Silva et al. showed that administration of vitamin C and vitamin D can reduce the severity of COVID-19 infection [31]. The findings were against those of Beran and Rawat. There are also several studies that focus on High-dose intravenous administration of Vitamin C (HDVC), which is another point for further discussion. Khokher et al., reported that HDVC has neither any significant effect on the death rate nor on the length of hospital stay in COVID-19 patients. The interesting finding was that HDVC could result in an increased length of ICU stay [27]. Consistently, Kwak’s study including eight studies on HDVC showed no efficacy of HDVC on mortality of COVID-19 patients [19].

The results of Ao’s study confirmed the former studies on IV vitamin C administration [16]. Of the 28 included studies in this review, only one study evaluated polyunsaturated Fatty Acids (PUFA). Mazidimoradi found that omega 3 PUFA reduces the severity of COVID-19 disease. They also discovered that individuals with COVID-19 have lower levels of omega 3 and omega 6 and with higher levels of omega 3 and omega 6, the chance of mechanical ventilation and hospitalization will be reduced [13].

Putting it all together, it appeared that only the administration of vitamin D has solid advantages in COVID-19 management. It attenuates the inflammatory markers and reduces the mortality and morbidity of the disease. In contrast, vitamin C did not show any benefits on the COVID-19 outcome. Other micronutrients including Zinc, vitamin A, vitamin B, etc. might have some benefits on the outcome of the disease but more investigations on these micronutrients are recommended.

The above findings could be used by physicians as a part of the COVID-19 patients' therapy, to reduce the morbidities, mortality, and duration of the disease. Although using the micronutrients as a part of treatment could reduce the need for prescribing high doses of other drugs, it requires close attention and monitoring of their dosage too. Besides their advantages, these micronutrients have also their side effects in case of overdose.

Our study selected 28 systematic reviews that included different study designs and populations to assess the effect of micronutrients on COVID-19 infection outcomes focusing on vitamin D and vitamin C as the most debated micronutrients for COVID-19. Although this is a great benefit of our study which evaluated the desirable number of studies with different populations and characteristics, consistencies among the findings of studies should raise our concern also about the limitations. Therefore, further investigations focusing on micronutrients with controversial findings, as well as other micronutrients in different populations considering the sociodemographic inequalities are recommended in the future.

Besides, in this challenging condition where there is an inconsistency between articles, it is also recommended to follow the guidelines which could be used as a reliable & helpful reference for treatment. As some other studies mentioned the importance of guideline preparation [44], we should know that guidelines could give physicians a better view with high certainty and confidence in therapy, especially in complicated cases.

One of the strengths of this article was how this study looked at having a joint approach including nutrition and diet with COVID-19, which was significantly associated with clinical outcomes related to COVID-19. This review also had limitations, such as the fact that despite the broad approach used in the literature search strategy of this study, all the necessary scientific literature on this topic may not have been included. Nevertheless, the data presented strongly suggest that health professionals with the necessary information can have a positive performance in managing proper nutrition for the best possible management of COVID-19.

## Conclusions

The COVID-19 pandemic caused the lifestyle, including nutrition and diet patterns, to go out of its natural rhythm, and in this context, the comprehensive management of complications has related to COVID-19, including the adequate intake of nutrients, from micronutrients to macronutrients are very important. In conclusion, among the different macronutrients and micronutrients that were used during the course of COVID-19, the administration of vitamin D has some advantages in COVID-19-infected patients. The dose of vitamin D used was highly variable among the studies from 1000 IU/day to 400,000 IU as a bolus within a few hours from diagnosis of COVID-19. Based on the findings, administration of vitamin D is helpful in reducing the morbidity and mortality and even shortening the length of hospitalization and there is no significant difference between different doses of vitamin D. However, it is vitamin D deficiency, which results in higher risk of morbidities and mortality. Also, vitamin D can reduce the severity of the disease and mortality rate by reducing the production of inflammatory cytokines. Therefore, its administration, especially in vitamin D deficient patients, is recommended. Despite all favorable debates, vitamin C appeared to have no efficacy in COVID-19 even in high doses. In the end, it is necessary to consider this point that although other micronutrients in this study showed benefits in the management of COVID-19, more research in this field is recommended to improve the nutritional conditions and lifestyle conditions of people in the management of COVID-19.

### Supplementary Information

**Additional file 1**. The search Keywords in the databases.

## Data Availability

The authors stated that all information provided in this article could be shared."Esmaeil Mehraeen" had full access to all of the data in this study and takes complete responsibility for the integrity of the data and the accuracy of the data analysis.
