# Supplementary material for: The effect of macronutrient and micronutrient supplements on COVID-19: an umbrella review
Source: J Health Popul Nutr. 2024 Jan 29;43:16. doi: 10.1186/s41043-024-00504-8 (PMC10826055; doi:10.1186/s41043-024-00504-8)
Supplement: Supplementary file 1 — Additional file 1. The search Keywords in the databases. [file 41043_2024_504_MOESM1_ESM.docx]

Keywords:
“COVID-19 Keywords”

MESH Terms:

COVID-19

SARS-CoV-2

Emtree Terms:

coronavirus disease 2019

severe acute respiratory syndrome coronavirus 2

SYNONYMS:

COVID-19

SARS-CoV-2

coronavirus disease 2019

severe acute respiratory syndrome coronavirus 2

SARS CoV 2

2019 Novel Coronavirus

2019-nCoV

2019 nCoV

Coronavirus Disease-19

Coronavirus Disease 19

SARS Coronavirus 2

Wuhan Seafood Market Pneumonia Virus

Wuhan Coronavirus

coronavirus disease 2

coronavirus disease 2019

coronavirus infection 2019

COVID

nCoV 2019

new coronavirus pneumonia

novel coronavirus

SARSCoV2

severe acute respiratory syndrome 2

severe acute respiratory syndrome coronavirus 2019 infection

severe acute respiratory syndrome CoV-2 infection

2019 new coronavirus

coronavirus SARS-2

HCoV-19

Human coronavirus 2019

nCoV-2019

novel 2019 coronavirus

SARS-2 (virus)

SARS-2-CoV

SARS-related coronavirus 2

SARS2 (virus)

Severe acute respiratory coronavirus 2

Severe acute respiratory syndrome 2

severe acute respiratory syndrome corona virus 2

severe acute respiratory syndrome coronavirus 2019

Severe acute respiratory syndrome coronoavirus 2

Severe acute respiratory syndrome coronvirus 2

severe acute respiratory syndrome CoV-2 virus

Severe acute respiratory syndrome related coronavirus 2

Severe acute respiratory syndrome virus 2

“Nutrient Keywords “

**Mesh Term :**

Nutrients

Trace Elements

Zinc

Iron

Micronutrients

Vitamins

Provitamins

Vitamin B Complex

Dietary Supplements

Prebiotics

Synbiotics

Probiotics

Dietary Proteins

Animal Proteins, Dietary

Dietary Carbohydrates

Dietary Fiber

Dietary Fats

Micronutrients [Pharmacological Action]

Trace Elements [Pharmacological Action]

24,25-Dihydroxyvitamin D 3

25-Hydroxyvitamin D 2

Acetylcarnitine

alpha-Tocopherol

Ascorbic Acid

beta Carotene

Beta-Cryptoxanthin

beta-Tocopherol

Biotin

Calcifediol

Calcitriol

Cholecalciferol

Cobamides

Dehydroascorbic Acid

Dehydrocholesterols

Dihydrotachysterol

Dihydroxycholecalciferols

Ergocalciferols

Ergosterol

Flavin Mononucleotide

Folic Acid

Formyltetrahydrofolates

Fursultiamin

gamma-Tocopherol

Hydroxocobalamin

Hydroxycholecalciferols

Inositol

Leucovorin

Niacin

Niacinamide

Nicotinic Acids

Palmitoylcarnitine

Pantothenic Acid

Pteroylpolyglutamic Acids

Pyridoxal

Pyridoxal Phosphate

Pyridoxamine

Pyridoxine

Riboflavin

sapotexanthin

Tetrahydrofolates

Thiamine

Thiamine Monophosphate

Thiamine Pyrophosphate

Thiamine Triphosphate

Thioctic Acid

Tocopherols

Tocotrienols

Vitamin A

Vitamin B 12

Vitamin B 6

Vitamin D

Vitamin E

Vitamin K

Vitamin K 1

Vitamin K 2

Vitamin K 3

Vitamin U

Boron

Chromium

Copper

Iodine

Iron

Manganese

Selenium

Sodium Selenite

Tin

Fatty Acids, Omega-3

Cod Liver Oil

Phosphorus, Dietary

Magnesium

Calcium

alpha-Tocopherol

Fatty Acids, Omega-6

Fatty Acids

**Emtree Terms:**

nutrient

trace element

trace metal

vitamin

Zinc

Iron

protein intake

carbohydrate intake

dietary supplement

provitamin

selenium

**Synonyms:**

Nutrient*

trace element*

trace metal*

Trace mineral*

vitamin*

Zinc

Iron

protein intake

carbohydrate intake

dietary supplement*

provitamin*

selenium

Micronutrient*

Prebiotic*

Synbiotic*

Probiotic*

Dietary Protein*

Dietary Carbohydrate*

Dietary Fiber*

Dietary Fat*

24,25-Dihydroxyvitamin D 3

25-Hydroxyvitamin D 2

Acetylcarnitine

alpha-Tocopherol

Ascorbic Acid

beta Carotene

Beta-Cryptoxanthin

beta-Tocopherol

Biotin

Calcifediol

Calcitriol

Cholecalciferol

Cobamides

Dehydroascorbic Acid

Dihydrotachysterol*

Dihydroxycholecalciferols

Ergocalciferols

Ergosterol

Flavin Mononucleotide

Folic Acid

Formyltetrahydrofolates

Fursultiamin

gamma-Tocopherol

Hydroxocobalamin

Hydroxycholecalciferols

Inositol

Leucovorin

Niacin

Niacinamide

Nicotinic Acids

Palmitoylcarnitine

Pantothenic Acid

Pteroylpolyglutamic Acids

Pyridoxal

Pyridoxamine

Pyridoxine

Riboflavin

sapotexanthin

Tetrahydrofolates

Thiamine

Thiamine Monophosphate

Thiamine Pyrophosphate

Thiamine Triphosphate

Thioctic Acid

Tocopherols

Tocotrienols

Boron

Chromium

Copper

Iodine

Manganese

Selenium

Sodium Selenite

Tin

Cod Liver Oil

Phosphorus, Dietary

Magnesium

Calcium

alpha-Tocopherol

Fatty Acids, Omega-6

Fatty Acid*

Esterified Fatty Acid*

Saturated Fatty Acid*

Aliphatic Acid*

Acid, Aliphatic

Omega-6 Fatty Acid*

Omega 6 Fatty Acid*

N-6 Fatty Acid*

N 6 Fatty Acid*

alpha Tocopherol

d-alpha Tocopherol

d alpha Tocopherol

R,R,R-alpha-Tocopherol

Tocopherol

d-alpha-Tocopheryl Acetate

Tocopheryl Acetate

alpha-Tocopheryl Calcium Succinate

alpha Tocopheryl Calcium Succinate

Pteroylglutamic Acid

Folvite

Folacin

Folate

Cyanocobalamin

Cobalamin*

Eritron

Dietary Phosphorus

Selenous Acid

Disodium Selenite

Sodium Selenite Pentahydrate

Monosodium Selenite

Selenite, Monosodium

Sodium Biselenite

Stannum

Macronutrient*

Biometal*

Neurobion

Dietary Supplementation*

Nutraceutical*

Nutriceutical*

Neutraceutical*

Prebiotic

Synbiotic

Probiotic

Dietary Protein*

Protein

Carbohydrate

Dietary Carbohydrate*

Dietary Fiber*

butaphosphan

cyanocobalamin

choline

dehydrocholic acid

magnesium orotate

orotic acid

ferrous

Tocovid

pentifyllin

nicotinic acid

cupric oxide

Omega 3 Fatty Acid*

Omega-3 Fatty Acid*

n-3 Oil

n 3 Oil

n3 Oil

n-3 Fatty Acid

n 3 Fatty Acid

n-3 PUFA

n 3 PUFA

n3 Fatty Acid

n3 PUFA

n3 Polyunsaturated Fatty Acid

n3 Oil*

n-3 Oil*

n 3 Oil

N-3 Fatty Acid

N 3 Fatty Acid

n-3 Polyunsaturated Fatty Acid

n 3 Polyunsaturated Fatty Acid

PubMed Search Query

Results : 268

(“COVID-19” [mesh] OR “SARS-CoV-2” [mesh] OR COVID-19[tiab] OR SARS-CoV-2[tiab] OR coronavirus disease 2019[tiab] OR severe acute respiratory syndrome coronavirus 2[tiab] OR SARS CoV 2 [tiab] OR 2019 Novel Coronavirus[tiab] OR 2019-nCoV [tiab] OR 2019 nCoV [tiab] OR Coronavirus Disease-19[tiab] OR Coronavirus Disease 19[tiab] OR SARS Coronavirus 2[tiab] OR Wuhan Seafood Market Pneumonia Virus[tiab] OR Wuhan Coronavirus[tiab] OR coronavirus disease 2[tiab] OR coronavirus disease 2019 [tiab] OR coronavirus infection 2019[tiab] OR COVID[tiab] OR nCoV 2019 [tiab] OR new coronavirus pneumonia[tiab] OR novel coronavirus [tiab] OR SARSCoV2 [tiab] OR severe acute respiratory syndrome 2[tiab] OR severe acute respiratory syndrome coronavirus 2019 infection[tiab] OR severe acute respiratory syndrome CoV-2 infection[tiab] OR 2019 new coronavirus[tiab] OR coronavirus SARS-2[tiab] OR HCoV-19[tiab] OR Human coronavirus 2019[tiab] OR nCoV-2019[tiab] OR novel 2019 coronavirus[tiab] OR SARS-2 (virus)[tiab] OR SARS-2-CoV[tiab] OR SARS-related coronavirus 2[tiab] OR SARS2 (virus)[tiab] OR Severe acute respiratory coronavirus 2[tiab] OR Severe acute respiratory syndrome 2 [tiab] OR severe acute respiratory syndrome corona virus 2[tiab] OR severe acute respiratory syndrome coronavirus 2019[tiab] OR Severe acute respiratory syndrome coronoavirus 2[tiab] OR Severe acute respiratory syndrome coronvirus 2[tiab] OR severe acute respiratory syndrome CoV-2 virus[tiab] OR Severe acute respiratory syndrome related coronavirus 2[tiab] OR Severe acute respiratory syndrome virus 2[tiab]) AND (“Nutrients”[mesh] OR “Trace Elements”[mesh] OR “Zinc”[mesh] OR “Iron”[mesh] OR “Micronutrients”[mesh] OR “Vitamins”[mesh] OR “Provitamins”[mesh] OR “Vitamin B Complex”[mesh] OR “Dietary Supplements”[mesh] OR “Prebiotics”[mesh] OR “Synbiotics”[mesh] OR “Probiotics”[mesh] OR “Dietary Proteins”[mesh] OR “Animal Proteins, Dietary”[mesh] OR “Dietary Carbohydrates”[mesh] OR “Dietary Fiber”[mesh] OR “Dietary Fats”[mesh] OR “Micronutrients [Pharmacological Action]”[mesh] OR “Trace Elements [Pharmacological Action]”[mesh] OR “24,25-Dihydroxyvitamin D 3”[mesh] OR “25-Hydroxyvitamin D 2”[mesh] OR “Acetylcarnitine”[mesh] OR “alpha-Tocopherol”[mesh] OR “Ascorbic Acid”[mesh] OR “beta Carotene”[mesh] OR “Beta-Cryptoxanthin”[mesh] OR “beta-Tocopherol”[mesh] OR “Biotin”[mesh] OR “Calcifediol”[mesh] OR “Calcitriol”[mesh] OR “Cholecalciferol”[mesh] OR “Cobamides”[mesh] OR “Dehydroascorbic Acid”[mesh] OR “Dehydrocholesterols”[mesh] OR “Dihydrotachysterol”[mesh] OR “Dihydroxycholecalciferols”[mesh] OR “Ergocalciferols”[mesh] OR “Ergosterol”[mesh] OR “Flavin Mononucleotide”[mesh] OR “Folic Acid”[mesh] OR “Formyltetrahydrofolates”[mesh] OR “Fursultiamin”[mesh] OR “gamma-Tocopherol”[mesh] OR “Hydroxocobalamin”[mesh] OR “Hydroxycholecalciferols”[mesh] OR “Inositol”[mesh] OR “Leucovorin”[mesh] OR “Niacin”[mesh] OR “Niacinamide”[mesh] OR “Nicotinic Acids”[mesh] OR “Palmitoylcarnitine”[mesh] OR “Pantothenic Acid”[mesh] OR “Pteroylpolyglutamic Acids”[mesh] OR “Pyridoxal”[mesh] OR “Pyridoxal Phosphate”[mesh] OR “Pyridoxamine”[mesh] OR “Pyridoxine”[mesh] OR “Riboflavin”[mesh] OR “sapotexanthin”[mesh] OR “Tetrahydrofolates”[mesh] OR “Thiamine”[mesh] OR “Thiamine Monophosphate”[mesh] OR “Thiamine Pyrophosphate”[mesh] OR “Thiamine Triphosphate”[mesh] OR “Thioctic Acid”[mesh] OR “Tocopherols”[mesh] OR “Tocotrienols”[mesh] OR “Vitamin A”[mesh] OR “Vitamin B 12”[mesh] OR “Vitamin B 6”[mesh] OR “Vitamin D”[mesh] OR “Vitamin E”[mesh] OR “Vitamin K”[mesh] OR “Vitamin K 1”[mesh] OR “Vitamin K 2”[mesh] OR “Vitamin K 3”[mesh] OR “Vitamin U”[mesh] OR “Boron”[mesh] OR “Chromium”[mesh] OR “Copper”[mesh] OR “Iodine”[mesh] OR “Iron”[mesh] OR “Manganese”[mesh] OR “Selenium”[mesh] OR “Sodium Selenite”[mesh] OR “Tin”[mesh] OR “Zinc”[mesh] OR “Fatty Acids, Omega-3”[mesh] OR “Cod Liver Oil”[mesh] OR “Phosphorus, Dietary”[mesh] OR “Magnesium”[mesh] OR “Calcium”[mesh] OR “alpha-Tocopherol”[mesh] OR “Fatty Acids, Omega-6”[mesh] OR “Fatty Acids”[mesh] OR Nutrient*[tiab] OR trace element*[tiab] OR trace metal*[tiab] OR Trace mineral*[tiab] OR vitamin*[tiab] OR Zinc[tiab] OR Iron[tiab] OR protein intake[tiab] OR carbohydrate intake[tiab] OR dietary supplement*[tiab] OR provitamin*[tiab] OR selenium[tiab] OR Micronutrient*[tiab] OR Prebiotic*[tiab] OR Synbiotic*[tiab] OR Probiotic*[tiab] OR Dietary Protein*[tiab] OR Dietary Carbohydrate*[tiab] OR Dietary Fiber*[tiab] OR Dietary Fat*[tiab] OR 24,25-Dihydroxyvitamin D 3[tiab] OR 25-Hydroxyvitamin D 2[tiab] OR Acetylcarnitine[tiab] OR alpha-Tocopherol[tiab] OR Ascorbic Acid[tiab] OR beta Carotene[tiab] OR Beta-Cryptoxanthin[tiab] OR beta-Tocopherol[tiab] OR Biotin[tiab] OR Calcifediol[tiab] OR Calcitriol[tiab] OR Cholecalciferol[tiab] OR Cobamides[tiab] OR Dehydroascorbic Acid[tiab] OR Dihydrotachysterol*[tiab] OR Dihydroxycholecalciferols[tiab] OR Ergocalciferols[tiab] OR Ergosterol[tiab] OR Flavin Mononucleotide[tiab] OR Folic Acid[tiab] OR Formyltetrahydrofolates[tiab] OR Fursultiamin[tiab] OR gamma-Tocopherol[tiab] OR Hydroxocobalamin[tiab] OR Hydroxycholecalciferols[tiab] OR Inositol[tiab] OR Leucovorin[tiab] OR Niacin[tiab] OR Niacinamide[tiab] OR Nicotinic Acids[tiab] OR Palmitoylcarnitine[tiab] OR Pantothenic Acid[tiab] OR Pteroylpolyglutamic Acids[tiab] OR Pyridoxal[tiab] OR Pyridoxamine[tiab] OR Pyridoxine[tiab] OR Riboflavin[tiab] OR sapotexanthin[tiab] OR Tetrahydrofolates[tiab] OR Thiamine[tiab] OR Thiamine Monophosphate[tiab] OR Thiamine Pyrophosphate[tiab] OR Thiamine Triphosphate[tiab] OR Thioctic Acid[tiab] OR Tocopherols[tiab] OR Tocotrienols[tiab] OR Boron[tiab] OR Chromium[tiab] OR Copper[tiab] OR Iodine[tiab] OR Manganese[tiab] OR Selenium[tiab] OR Sodium Selenite[tiab] OR Tin[tiab] OR Cod Liver Oil[tiab] OR Phosphorus, Dietary[tiab] OR Magnesium[tiab] OR Calcium[tiab] OR alpha-Tocopherol[tiab] OR Fatty Acids, Omega-6[tiab] OR Fatty Acid*[tiab] OR Esterified Fatty Acid*[tiab] OR Saturated Fatty Acid*[tiab] OR Aliphatic Acid*[tiab] OR Acid, Aliphatic[tiab] OR Omega-6 Fatty Acid*[tiab] OR Omega 6 Fatty Acid*[tiab] OR N-6 Fatty Acid*[tiab] OR N 6 Fatty Acid*[tiab] OR alpha Tocopherol[tiab] OR d-alpha Tocopherol[tiab] OR d alpha Tocopherol[tiab] OR R,R,R-alpha-Tocopherol[tiab] OR Tocopherol[tiab] OR d-alpha-Tocopheryl Acetate[tiab] OR Tocopheryl Acetate[tiab] OR alpha-Tocopheryl Calcium Succinate[tiab] OR alpha Tocopheryl Calcium Succinate[tiab] OR Pteroylglutamic Acid[tiab] OR Folvite[tiab] OR Folacin[tiab] OR Folate[tiab] OR Cyanocobalamin[tiab] OR Cobalamin*[tiab] OR Eritron[tiab] OR Dietary Phosphorus[tiab] OR Selenous Acid[tiab] OR Disodium Selenite[tiab] OR Sodium Selenite Pentahydrate[tiab] OR Monosodium Selenite[tiab] OR Selenite, Monosodium[tiab] OR Sodium Biselenite[tiab] OR Stannum[tiab] OR Macronutrient*[tiab] OR Biometal*[tiab] OR Neurobion[tiab] OR Dietary Supplementation*[tiab] OR Nutraceutical*[tiab] OR Nutriceutical*[tiab] OR Neutraceutical*[tiab] OR Prebiotic[tiab] OR Synbiotic[tiab] OR Probiotic[tiab] OR Dietary Protein*[tiab] OR Protein[tiab] OR Carbohydrate[tiab] OR Dietary Carbohydrate*[tiab] OR Dietary Fiber*[tiab] OR butaphosphan[tiab] OR cyanocobalamin[tiab] OR choline[tiab] OR dehydrocholic acid[tiab] OR magnesium orotate[tiab] OR orotic acid[tiab] OR ferrous[tiab] OR Tocovid[tiab] OR pentifyllin[tiab] OR nicotinic acid[tiab] OR cupric oxide[tiab] OR Omega 3 Fatty Acid*[tiab] OR Omega-3 Fatty Acid*[tiab] OR n-3 Oil[tiab] OR n 3 Oil[tiab] OR n3 Oil[tiab] OR n-3 Fatty Acid[tiab] OR n 3 Fatty Acid[tiab] OR n-3 PUFA[tiab] OR n 3 PUFA[tiab] OR n3 Fatty Acid[tiab] OR n3 PUFA[tiab] OR n3 Polyunsaturated Fatty Acid[tiab] OR n3 Oil*[tiab] OR n-3 Oil*[tiab] OR n 3 Oil[tiab] OR N-3 Fatty Acid[tiab] OR N 3 Fatty Acid[tiab] OR n-3 Polyunsaturated Fatty Acid[tiab] OR n 3 Polyunsaturated Fatty Acid[tiab]) AND (systematic review[tiab] OR meta analysis[tiab] OR Meta-analysis[tiab] OR systematic-review[tiab])

Embase Search Query

Results : 527

(’coronavirus disease 2019’/exp OR ’severe acute respiratory syndrome coronavirus 2’/exp OR ’COVID-19’:ab,ti OR ’SARS-CoV-2’:ab,ti OR ’coronavirus disease 2019’:ab,ti OR ’severe acute respiratory syndrome coronavirus 2’:ab,ti OR ’SARS CoV 2 ‘:ab,ti OR ’2019 Novel Coronavirus’:ab,ti OR ’2019-nCoV ‘:ab,ti OR ’2019 nCoV ‘:ab,ti OR ’Coronavirus Disease-19’:ab,ti OR ’Coronavirus Disease 19’:ab,ti OR ’SARS Coronavirus 2’:ab,ti OR ’Wuhan Seafood Market Pneumonia Virus’:ab,ti OR ’Wuhan Coronavirus’:ab,ti OR ’coronavirus disease 2’:ab,ti OR ’coronavirus disease 2019 ‘:ab,ti OR ’coronavirus infection 2019’:ab,ti OR ’COVID’:ab,ti OR ’nCoV 2019 ‘:ab,ti OR ’new coronavirus pneumonia’:ab,ti OR ’novel coronavirus ‘:ab,ti OR ’SARSCoV2 ‘:ab,ti OR ’severe acute respiratory syndrome 2’:ab,ti OR ’severe acute respiratory syndrome coronavirus 2019 infection’:ab,ti OR ’severe acute respiratory syndrome CoV-2 infection’:ab,ti OR ’2019 new coronavirus’:ab,ti OR ’coronavirus SARS-2’:ab,ti OR ’HCoV-19’:ab,ti OR ’Human coronavirus 2019’:ab,ti OR ’nCoV-2019’:ab,ti OR ’novel 2019 coronavirus’:ab,ti OR ’SARS-2 (virus)’:ab,ti OR ’SARS-2-CoV’:ab,ti OR ’SARS-related coronavirus 2’:ab,ti OR ’SARS2 (virus)’:ab,ti OR ’Severe acute respiratory coronavirus 2’:ab,ti OR ’Severe acute respiratory syndrome 2 ‘:ab,ti OR ’severe acute respiratory syndrome corona virus 2’:ab,ti OR ’severe acute respiratory syndrome coronavirus 2019’:ab,ti OR ’Severe acute respiratory syndrome coronoavirus 2’:ab,ti OR ’Severe acute respiratory syndrome coronvirus 2’:ab,ti OR ’severe acute respiratory syndrome CoV-2 virus’:ab,ti OR ’Severe acute respiratory syndrome related coronavirus 2’:ab,ti OR ’Severe acute respiratory syndrome virus 2’:ab,ti) AND (‘nutrient’/exp OR ‘trace element’/exp OR ‘trace metal’/exp OR ‘vitamin’/exp OR ‘Zinc’/exp OR ‘Iron’/exp OR ‘protein intake’/exp OR ‘carbohydrate intake’/exp OR ‘dietary supplement’/exp OR ‘provitamin’/exp OR ‘selenium’/exp OR ‘Nutrient*’:ti,ab OR ‘trace element*’:ti,ab OR ‘trace metal*’:ti,ab OR ‘Trace mineral*’:ti,ab OR ‘vitamin*’:ti,ab OR ‘Zinc’:ti,ab OR ‘Iron’:ti,ab OR ‘protein intake’:ti,ab OR ‘carbohydrate intake’:ti,ab OR ‘dietary supplement*’:ti,ab OR ‘provitamin*’:ti,ab OR ‘selenium’:ti,ab OR ‘Micronutrient*’:ti,ab OR ‘Prebiotic*’:ti,ab OR ‘Synbiotic*’:ti,ab OR ‘Probiotic*’:ti,ab OR ‘Dietary Protein*’:ti,ab OR ‘Dietary Carbohydrate*’:ti,ab OR ‘Dietary Fiber*’:ti,ab OR ‘Dietary Fat*’:ti,ab OR ‘24,25-Dihydroxyvitamin D 3’:ti,ab OR ‘25-Hydroxyvitamin D 2’:ti,ab OR ‘Acetylcarnitine’:ti,ab OR ‘alpha-Tocopherol’:ti,ab OR ‘Ascorbic Acid’:ti,ab OR ‘beta Carotene’:ti,ab OR ‘Beta-Cryptoxanthin’:ti,ab OR ‘beta-Tocopherol’:ti,ab OR ‘Biotin’:ti,ab OR ‘Calcifediol’:ti,ab OR ‘Calcitriol’:ti,ab OR ‘Cholecalciferol’:ti,ab OR ‘Cobamides’:ti,ab OR ‘Dehydroascorbic Acid’:ti,ab OR ‘Dihydrotachysterol*’:ti,ab OR ‘Dihydroxycholecalciferols’:ti,ab OR ‘Ergocalciferols’:ti,ab OR ‘Ergosterol’:ti,ab OR ‘Flavin Mononucleotide’:ti,ab OR ‘Folic Acid’:ti,ab OR ‘Formyltetrahydrofolates’:ti,ab OR ‘Fursultiamin’:ti,ab OR ‘gamma-Tocopherol’:ti,ab OR ‘Hydroxocobalamin’:ti,ab OR ‘Hydroxycholecalciferols’:ti,ab OR ‘Inositol’:ti,ab OR ‘Leucovorin’:ti,ab OR ‘Niacin’:ti,ab OR ‘Niacinamide’:ti,ab OR ‘Nicotinic Acids’:ti,ab OR ‘Palmitoylcarnitine’:ti,ab OR ‘Pantothenic Acid’:ti,ab OR ‘Pteroylpolyglutamic Acids’:ti,ab OR ‘Pyridoxal’:ti,ab OR ‘Pyridoxamine’:ti,ab OR ‘Pyridoxine’:ti,ab OR ‘Riboflavin’:ti,ab OR ‘sapotexanthin’:ti,ab OR ‘Tetrahydrofolates’:ti,ab OR ‘Thiamine’:ti,ab OR ‘Thiamine Monophosphate’:ti,ab OR ‘Thiamine Pyrophosphate’:ti,ab OR ‘Thiamine Triphosphate’:ti,ab OR ‘Thioctic Acid’:ti,ab OR ‘Tocopherols’:ti,ab OR ‘Tocotrienols’:ti,ab OR ‘Boron’:ti,ab OR ‘Chromium’:ti,ab OR ‘Copper’:ti,ab OR ‘Iodine’:ti,ab OR ‘Manganese’:ti,ab OR ‘Selenium’:ti,ab OR ‘Sodium Selenite’:ti,ab OR ‘Tin’:ti,ab OR ‘Cod Liver Oil’:ti,ab OR ‘Phosphorus, Dietary’:ti,ab OR ‘Magnesium’:ti,ab OR ‘Calcium’:ti,ab OR ‘alpha-Tocopherol’:ti,ab OR ‘Fatty Acids, Omega-6’:ti,ab OR ‘Fatty Acid*’:ti,ab OR ‘Esterified Fatty Acid*’:ti,ab OR ‘Saturated Fatty Acid*’:ti,ab OR ‘Aliphatic Acid*’:ti,ab OR ‘Acid, Aliphatic’:ti,ab OR ‘Omega-6 Fatty Acid*’:ti,ab OR ‘Omega 6 Fatty Acid*’:ti,ab OR ‘N-6 Fatty Acid*’:ti,ab OR ‘N 6 Fatty Acid*’:ti,ab OR ‘alpha Tocopherol’:ti,ab OR ‘d-alpha Tocopherol’:ti,ab OR ‘d alpha Tocopherol’:ti,ab OR ‘R,R,R-alpha-Tocopherol’:ti,ab OR ‘Tocopherol’:ti,ab OR ‘d-alpha-Tocopheryl Acetate’:ti,ab OR ‘Tocopheryl Acetate’:ti,ab OR ‘alpha-Tocopheryl Calcium Succinate’:ti,ab OR ‘alpha Tocopheryl Calcium Succinate’:ti,ab OR ‘Pteroylglutamic Acid’:ti,ab OR ‘Folvite’:ti,ab OR ‘Folacin’:ti,ab OR ‘Folate’:ti,ab OR ‘Cyanocobalamin’:ti,ab OR ‘Cobalamin*’:ti,ab OR ‘Eritron’:ti,ab OR ‘Dietary Phosphorus’:ti,ab OR ‘Selenous Acid’:ti,ab OR ‘Disodium Selenite’:ti,ab OR ‘Sodium Selenite Pentahydrate’:ti,ab OR ‘Monosodium Selenite’:ti,ab OR ‘Selenite, Monosodium’:ti,ab OR ‘Sodium Biselenite’:ti,ab OR ‘Stannum’:ti,ab OR ‘Macronutrient*’:ti,ab OR ‘Biometal*’:ti,ab OR ‘Neurobion’:ti,ab OR ‘Dietary Supplementation*’:ti,ab OR ‘Nutraceutical*’:ti,ab OR ‘Nutriceutical*’:ti,ab OR ‘Neutraceutical*’:ti,ab OR ‘Prebiotic’:ti,ab OR ‘Synbiotic’:ti,ab OR ‘Probiotic’:ti,ab OR ‘Dietary Protein*’:ti,ab OR ‘Protein’:ti,ab OR ‘Carbohydrate’:ti,ab OR ‘Dietary Carbohydrate*’:ti,ab OR ‘Dietary Fiber*’:ti,ab OR ‘butaphosphan’:ti,ab OR ‘cyanocobalamin’:ti,ab OR ‘choline’:ti,ab OR ‘dehydrocholic acid’:ti,ab OR ‘magnesium orotate’:ti,ab OR ‘orotic acid’:ti,ab OR ‘ferrous’:ti,ab OR ‘Tocovid’:ti,ab OR ‘pentifyllin’:ti,ab OR ‘nicotinic acid’:ti,ab OR ‘cupric oxide’:ti,ab OR ‘Omega 3 Fatty Acid*’:ti,ab OR ‘Omega-3 Fatty Acid*’:ti,ab OR ‘n-3 Oil’:ti,ab OR ‘n 3 Oil’:ti,ab OR ‘n3 Oil’:ti,ab OR ‘n-3 Fatty Acid’:ti,ab OR ‘n 3 Fatty Acid’:ti,ab OR ‘n-3 PUFA’:ti,ab OR ‘n 3 PUFA’:ti,ab OR ‘n3 Fatty Acid’:ti,ab OR ‘n3 PUFA’:ti,ab OR ‘n3 Polyunsaturated Fatty Acid’:ti,ab OR ‘n3 Oil*’:ti,ab OR ‘n-3 Oil*’:ti,ab OR ‘n 3 Oil’:ti,ab OR ‘N-3 Fatty Acid’:ti,ab OR ‘N 3 Fatty Acid’:ti,ab OR ‘n-3 Polyunsaturated Fatty Acid’:ti,ab OR ‘n 3 Polyunsaturated Fatty Acid’:ti,ab) AND (‘systematic review’:ti,ab OR ‘meta analysis’:ti,ab OR ‘Meta-analysis’:ti,ab OR ‘systematic-review’:ti,ab)

Scopus Search Query

Results : 1490

(TITLE-ABS-KEY (“COVID-19” OR “SARS-CoV-2” OR “coronavirus disease 2019” OR “severe acute respiratory syndrome coronavirus 2” OR “SARS CoV 2 “ OR “2019 Novel Coronavirus” OR “2019-nCoV “ OR “2019 nCoV “ OR “Coronavirus Disease-19” OR “Coronavirus Disease 19” OR “SARS Coronavirus 2” OR “Wuhan Seafood Market Pneumonia Virus” OR “Wuhan Coronavirus” OR “coronavirus disease 2” OR “coronavirus disease 2019 “ OR “coronavirus infection 2019” OR “COVID” OR “nCoV 2019 “ OR “new coronavirus pneumonia” OR “novel coronavirus “ OR “SARSCoV2 “ OR “severe acute respiratory syndrome 2” OR “severe acute respiratory syndrome coronavirus 2019 infection” OR “severe acute respiratory syndrome CoV-2 infection” OR “2019 new coronavirus” OR “coronavirus SARS-2” OR “HCoV-19” OR “Human coronavirus 2019” OR “nCoV-2019” OR “novel 2019 coronavirus” OR “SARS-2 (virus)” OR “SARS-2-CoV” OR “SARS-related coronavirus 2” OR “SARS2 (virus)” OR “Severe acute respiratory coronavirus 2” OR “Severe acute respiratory syndrome 2 “ OR “severe acute respiratory syndrome corona virus 2” OR “severe acute respiratory syndrome coronavirus 2019” OR “Severe acute respiratory syndrome coronoavirus 2” OR “Severe acute respiratory syndrome coronvirus 2” OR “severe acute respiratory syndrome CoV-2 virus” OR “Severe acute respiratory syndrome related coronavirus 2” OR “Severe acute respiratory syndrome virus 2”)) AND (TITLE-ABS-KEY (“Nutrient*” OR “trace element*” OR “trace metal*” OR “Trace mineral*” OR “vitamin*” OR “Zinc” OR “Iron” OR “protein intake” OR “carbohydrate intake” OR “dietary supplement*” OR “provitamin*” OR “selenium” OR “Micronutrient*” OR “Prebiotic*” OR “Synbiotic*” OR “Probiotic*” OR “Dietary Protein*” OR “Dietary Carbohydrate*” OR “Dietary Fiber*” OR “Dietary Fat*” OR “24,25-Dihydroxyvitamin D 3” OR “25-Hydroxyvitamin D 2” OR “Acetylcarnitine” OR “alpha-Tocopherol” OR “Ascorbic Acid” OR “beta Carotene” OR “Beta-Cryptoxanthin” OR “beta-Tocopherol” OR “Biotin” OR “Calcifediol” OR “Calcitriol” OR “Cholecalciferol” OR “Cobamides” OR “Dehydroascorbic Acid” OR “Dihydrotachysterol*” OR “Dihydroxycholecalciferols” OR “Ergocalciferols” OR “Ergosterol” OR “Flavin Mononucleotide” OR “Folic Acid” OR “Formyltetrahydrofolates” OR “Fursultiamin” OR “gamma-Tocopherol” OR “Hydroxocobalamin” OR “Hydroxycholecalciferols” OR “Inositol” OR “Leucovorin” OR “Niacin” OR “Niacinamide” OR “Nicotinic Acids” OR “Palmitoylcarnitine” OR “Pantothenic Acid” OR “Pteroylpolyglutamic Acids” OR “Pyridoxal” OR “Pyridoxamine” OR “Pyridoxine” OR “Riboflavin” OR “sapotexanthin” OR “Tetrahydrofolates” OR “Thiamine” OR “Thiamine Monophosphate” OR “Thiamine Pyrophosphate” OR “Thiamine Triphosphate” OR “Thioctic Acid” OR “Tocopherols” OR “Tocotrienols” OR “Boron” OR “Chromium” OR “Copper” OR “Iodine” OR “Manganese” OR “Selenium” OR “Sodium Selenite” OR “Tin” OR “Cod Liver Oil” OR “Phosphorus, Dietary” OR “Magnesium” OR “Calcium” OR “alpha-Tocopherol” OR “Fatty Acids, Omega-6” OR “Fatty Acid*” OR “Esterified Fatty Acid*” OR “Saturated Fatty Acid*” OR “Aliphatic Acid*” OR “Acid, Aliphatic” OR “Omega-6 Fatty Acid*” OR “Omega 6 Fatty Acid*” OR “N-6 Fatty Acid*” OR “N 6 Fatty Acid*” OR “alpha Tocopherol” OR “d-alpha Tocopherol” OR “d alpha Tocopherol” OR “R,R,R-alpha-Tocopherol” OR “Tocopherol” OR “d-alpha-Tocopheryl Acetate” OR “Tocopheryl Acetate” OR “alpha-Tocopheryl Calcium Succinate” OR “alpha Tocopheryl Calcium Succinate” OR “Pteroylglutamic Acid” OR “Folvite” OR “Folacin” OR “Folate” OR “Cyanocobalamin” OR “Cobalamin*” OR “Eritron” OR “Dietary Phosphorus” OR “Selenous Acid” OR “Disodium Selenite” OR “Sodium Selenite Pentahydrate” OR “Monosodium Selenite” OR “Selenite, Monosodium” OR “Sodium Biselenite” OR “Stannum” OR “Macronutrient*” OR “Biometal*” OR “Neurobion” OR “Dietary Supplementation*” OR “Nutraceutical*” OR “Nutriceutical*” OR “Neutraceutical*” OR “Prebiotic” OR “Synbiotic” OR “Probiotic” OR “Dietary Protein*” OR “Protein” OR “Carbohydrate” OR “Dietary Carbohydrate*” OR “Dietary Fiber*” OR “butaphosphan” OR “cyanocobalamin” OR “choline” OR “dehydrocholic acid” OR “magnesium orotate” OR “orotic acid” OR “ferrous” OR “Tocovid” OR “pentifyllin” OR “nicotinic acid” OR “cupric oxide” OR “Omega 3 Fatty Acid*” OR “Omega-3 Fatty Acid*” OR “n-3 Oil” OR “n 3 Oil” OR “n3 Oil” OR “n-3 Fatty Acid” OR “n 3 Fatty Acid” OR “n-3 PUFA” OR “n 3 PUFA” OR “n3 Fatty Acid” OR “n3 PUFA” OR “n3 Polyunsaturated Fatty Acid” OR “n3 Oil*” OR “n-3 Oil*” OR “n 3 Oil” OR “N-3 Fatty Acid” OR “N 3 Fatty Acid” OR “n-3 Polyunsaturated Fatty Acid” OR “n 3 Polyunsaturated Fatty Acid”)) AND (TITLE-ABS-KEY ("systematic review" OR "meta analysis" OR "Meta-analysis"))

Web of science Search Query

Results : 444

(TS= (“COVID-19” OR “SARS-CoV-2” OR “coronavirus disease 2019” OR “severe acute respiratory syndrome coronavirus 2” OR “SARS CoV 2 “ OR “2019 Novel Coronavirus” OR “2019-nCoV “ OR “2019 nCoV “ OR “Coronavirus Disease-19” OR “Coronavirus Disease 19” OR “SARS Coronavirus 2” OR “Wuhan Seafood Market Pneumonia Virus” OR “Wuhan Coronavirus” OR “coronavirus disease 2” OR “coronavirus disease 2019 “ OR “coronavirus infection 2019” OR “COVID” OR “nCoV 2019 “ OR “new coronavirus pneumonia” OR “novel coronavirus “ OR “SARSCoV2 “ OR “severe acute respiratory syndrome 2” OR “severe acute respiratory syndrome coronavirus 2019 infection” OR “severe acute respiratory syndrome CoV-2 infection” OR “2019 new coronavirus” OR “coronavirus SARS-2” OR “HCoV-19” OR “Human coronavirus 2019” OR “nCoV-2019” OR “novel 2019 coronavirus” OR “SARS-2 (virus)” OR “SARS-2-CoV” OR “SARS-related coronavirus 2” OR “SARS2 (virus)” OR “Severe acute respiratory coronavirus 2” OR “Severe acute respiratory syndrome 2 “ OR “severe acute respiratory syndrome corona virus 2” OR “severe acute respiratory syndrome coronavirus 2019” OR “Severe acute respiratory syndrome coronoavirus 2” OR “Severe acute respiratory syndrome coronvirus 2” OR “severe acute respiratory syndrome CoV-2 virus” OR “Severe acute respiratory syndrome related coronavirus 2” OR “Severe acute respiratory syndrome virus 2”)) AND (TS= (“Nutrient*” OR “trace element*” OR “trace metal*” OR “Trace mineral*” OR “vitamin*” OR “Zinc” OR “Iron” OR “protein intake” OR “carbohydrate intake” OR “dietary supplement*” OR “provitamin*” OR “selenium” OR “Micronutrient*” OR “Prebiotic*” OR “Synbiotic*” OR “Probiotic*” OR “Dietary Protein*” OR “Dietary Carbohydrate*” OR “Dietary Fiber*” OR “Dietary Fat*” OR “24,25-Dihydroxyvitamin D 3” OR “25-Hydroxyvitamin D 2” OR “Acetylcarnitine” OR “alpha-Tocopherol” OR “Ascorbic Acid” OR “beta Carotene” OR “Beta-Cryptoxanthin” OR “beta-Tocopherol” OR “Biotin” OR “Calcifediol” OR “Calcitriol” OR “Cholecalciferol” OR “Cobamides” OR “Dehydroascorbic Acid” OR “Dihydrotachysterol*” OR “Dihydroxycholecalciferols” OR “Ergocalciferols” OR “Ergosterol” OR “Flavin Mononucleotide” OR “Folic Acid” OR “Formyltetrahydrofolates” OR “Fursultiamin” OR “gamma-Tocopherol” OR “Hydroxocobalamin” OR “Hydroxycholecalciferols” OR “Inositol” OR “Leucovorin” OR “Niacin” OR “Niacinamide” OR “Nicotinic Acids” OR “Palmitoylcarnitine” OR “Pantothenic Acid” OR “Pteroylpolyglutamic Acids” OR “Pyridoxal” OR “Pyridoxamine” OR “Pyridoxine” OR “Riboflavin” OR “sapotexanthin” OR “Tetrahydrofolates” OR “Thiamine” OR “Thiamine Monophosphate” OR “Thiamine Pyrophosphate” OR “Thiamine Triphosphate” OR “Thioctic Acid” OR “Tocopherols” OR “Tocotrienols” OR “Boron” OR “Chromium” OR “Copper” OR “Iodine” OR “Manganese” OR “Selenium” OR “Sodium Selenite” OR “Tin” OR “Cod Liver Oil” OR “Phosphorus, Dietary” OR “Magnesium” OR “Calcium” OR “alpha-Tocopherol” OR “Fatty Acids, Omega-6” OR “Fatty Acid*” OR “Esterified Fatty Acid*” OR “Saturated Fatty Acid*” OR “Aliphatic Acid*” OR “Acid, Aliphatic” OR “Omega-6 Fatty Acid*” OR “Omega 6 Fatty Acid*” OR “N-6 Fatty Acid*” OR “N 6 Fatty Acid*” OR “alpha Tocopherol” OR “d-alpha Tocopherol” OR “d alpha Tocopherol” OR “R,R,R-alpha-Tocopherol” OR “Tocopherol” OR “d-alpha-Tocopheryl Acetate” OR “Tocopheryl Acetate” OR “alpha-Tocopheryl Calcium Succinate” OR “alpha Tocopheryl Calcium Succinate” OR “Pteroylglutamic Acid” OR “Folvite” OR “Folacin” OR “Folate” OR “Cyanocobalamin” OR “Cobalamin*” OR “Eritron” OR “Dietary Phosphorus” OR “Selenous Acid” OR “Disodium Selenite” OR “Sodium Selenite Pentahydrate” OR “Monosodium Selenite” OR “Selenite, Monosodium” OR “Sodium Biselenite” OR “Stannum” OR “Macronutrient*” OR “Biometal*” OR “Neurobion” OR “Dietary Supplementation*” OR “Nutraceutical*” OR “Nutriceutical*” OR “Neutraceutical*” OR “Prebiotic” OR “Synbiotic” OR “Probiotic” OR “Dietary Protein*” OR “Protein” OR “Carbohydrate” OR “Dietary Carbohydrate*” OR “Dietary Fiber*” OR “butaphosphan” OR “cyanocobalamin” OR “choline” OR “dehydrocholic acid” OR “magnesium orotate” OR “orotic acid” OR “ferrous” OR “Tocovid” OR “pentifyllin” OR “nicotinic acid” OR “cupric oxide” OR “Omega 3 Fatty Acid*” OR “Omega-3 Fatty Acid*” OR “n-3 Oil” OR “n 3 Oil” OR “n3 Oil” OR “n-3 Fatty Acid” OR “n 3 Fatty Acid” OR “n-3 PUFA” OR “n 3 PUFA” OR “n3 Fatty Acid” OR “n3 PUFA” OR “n3 Polyunsaturated Fatty Acid” OR “n3 Oil*” OR “n-3 Oil*” OR “n 3 Oil” OR “N-3 Fatty Acid” OR “N 3 Fatty Acid” OR “n-3 Polyunsaturated Fatty Acid” OR “n 3 Polyunsaturated Fatty Acid”)) AND (TS= ("systematic review" OR "meta analysis" OR "Meta-analysis" OR “systematic” OR “systematic-review”
